# Increased Complement C1q Level Marks Active Disease in Human Tuberculosis

**DOI:** 10.1371/journal.pone.0092340

**Published:** 2014-03-19

**Authors:** Yi Cai, Qianting Yang, Yueqiang Tang, Mingxia Zhang, Haiying Liu, Guoliang Zhang, Qunyi Deng, Jian Huang, Zhiliang Gao, Boping Zhou, Carl G. Feng, Xinchun Chen

**Affiliations:** 1 Department of Infectious Diseases, The Third Affiliated Hospital of Sun Yat-Sen University, Guangzhou, China; 2 Guangdong Key Laboratory for Emerging Infectious Diseases, Shenzhen Third People's Hospital, Shenzhen, China; 3 Shenzhen Key Laboratory of Infection and Immunity, Shenzhen Third People's Hospital, Guangdong Medical College, Shenzhen, China; 4 Institute of Pathogen Biology, Chinese Academy of Medical Sciences, Beijing, China; 5 Shanghai-MOST Key Laboratory of Disease and Health Genomics, National Engineering Center for Biochip at Shanghai, Shanghai, China; 6 Department of Infectious Diseases and Immunology, Sydney Medical School, The University of Sydney University, Australia; Hopital Raymond Poincare - Universite Versailles St. Quentin, France

## Abstract

**Background:**

Complement functions as an important host defense system and complement C5 and C7 have been implicated in immunopathology of tuberculosis. However, little is known about the role of other complement components in tuberculosis.

**Methods:**

Complement gene expression in peripheral blood mononuclear cells of tuberculosis patients and controls were determined using whole genome transcriptional microarray assays. The mRNA and protein levels of three C1q components, C1qA, C1qB, and C1qC, were further validated by qRT-PCR and enzyme-linked immunosorbent assay, respectively. The percentages of C1q expression in CD14 positive cells were determined by flow cytometry. Finally, C1qC protein level was quantified in the pleural fluid of tuberculosis and non-tuberculosis pleurisy.

**Results:**

C1q expression increases significantly in the peripheral blood of patients with active tuberculosis compared to healthy controls and individuals with latent TB infection. The percentage of C1q-expressing CD14 positive cells is significantly increased in active TB patients. C1q expression in the peripheral blood correlates with sputum smear positivity in tuberculosis patients and is reduced after anti-tuberculosis chemotherapy. Notably, receiver operating characteristic analysis showed that C1qC mRNA levels in peripheral blood efficiently discriminate active from latent tuberculosis infection and healthy controls. Additionally, C1qC protein level in pleural effusion shows improved power in discriminating tuberculosis from non-tuberculosis pleurisy when compared to other inflammatory markers, such as IL-6 and TNF-α.

**Conclusions:**

C1q expression correlates with active disease in human tuberculosis. C1q could be a potential diagnostic marker to discriminate active tuberculosis from latent tuberculosis infection as well as tuberculosis pleurisy from non-tuberculosis pleurisy.

## Introduction

Despite the availability of reasonably inexpensive and well-tolerated chemotherapy, tuberculosis (TB) continues to be a major global health problem, causing an estimated 8.6 million new cases and 1.3 million deaths in 2012 [1]. Current BCG vaccine provides good protection against severe childhood TB, but is less useful in the prevention of adult TB because of its protection provided against TB in adults is very variable [2], [3]. In addition to effective vaccines, a rapid and accurate diagnosis test for active TB will prompt early treatment and reduce transmission thereby facilitating TB control. Unfortunately, the available diagnostic tests have significant shortcomings, such as lack of sensitivity and specificity [4]. This is particularly problematic in the settings of extra-pulmonary, sputum-smear negative and pediatric TB. To overcome these shortcomings, an Interferon-γ (IFN-γ) release assays (IGRAs) has been developed to identify individuals with latent TB infection (LTBI) [5], [6], [7], [8], [9]. However, meta-analysis showed that IGRAs had limited power in distinguishing active TB from LTBI, although it was useful for identifying subjects with *Mycobacterium tuberculosis* (Mtb) infection from those without infection [10], [11]. As a consequence, identification of biomarkers that can accurately distinguish individuals with active TB from LTBI has been designated an important research priority in TB research [12].

Complement functions as an important host defense system that senses danger signals triggered by pathogen- and host tissue damage-associated pattern molecules [13], [14], [15]. In this regard, both complement C5 and C7; have been implicated in regulating immunopathology of TB [16]. However, little is known about the role of other complement components in TB.

C1q is a 460-kDa protein composed of 18 polypeptide chains (6A, 6B and 6C). C1q is synthesized by monocyte/macrophage lineage [17], [18], DCs [19], microglia [20] and other cell types [21]. Macrophages are abundant in tissues and are probably the major source of blood C1q [22]. It is reported that the microbial factors and inflammatory cytokines can regulate C1q production. LPS, peptidoglycan (PGN), zymosan, IFN-γ and IL-6 increase but IL-1 inhibits C1q production by macrophages [22]. A classical function of C1q is to initiate complement activation [23]. Besides complement activation, C1q has been recently recognized to have versatile non-complement activation functions [14]. It is reported that C1q can modulate dendritic cell maturation, pro-inflammatory cytokine production, and T- and B-cell responses [14], [24], [25], [26], [27], [28]. In a recent report comparing whole blood microarray data from LTBI and TB patients C1q mRNA was differentially expressed [29]. To investigate the role of C1q in human TB, we have defined the mRNA and protein expression pattern of C1q in the peripheral blood and the site of infection. We report that elevated C1q expression is strongly associated with active TB disease and disease severity. C1q is highly enriched at the site of disease compared to the peripheral blood. More importantly, C1qC is a useful biomarker for diagnosis of active TB.

## Methods

### Ethics Statement

This work received approval from the Institutional Review Board of Shenzhen Third People's Hospital. Written informed consent was obtained from all participants.

### Subjects and Samples

A total of three cohorts of participants including one microarray discovery set, one validation cohort, and one pleurisy patient cohort were recruited from May 2011 to December 2012 at Shenzhen Third People's Hospital in this study. Each cohort consists of active tuberculosis patients and controls recruited for the comparison; the detailed demographic characteristics are listed in Table 1. Diagnosis of active TB was based on clinical symptoms, chest radiography, microscopy for acid fast bacilli (AFB), sputum Mtb culture and response to anti-TB chemotherapy. All sputum specimens were digested and decontaminated of other bacteria by the standard N-acetyl-L-cysteine (NALC)-NaOH-sodium citrate method. An aliquot of the specimen was used for microscopical examination of Ziehl-Neelsen stained smears and the remainder was used for parallel testing with BACTEC TB 960 culture system as per the manufacturer's instructions. Sputum samples were classified according to the highest number of AFB per specimen. Previously established Mtb specific IGRAs were used to differentiate individuals with LTBI from healthy controls without infection [9]. The diagnosis of TB pleurisy was based on pleural fluid and/or biopsy cultures or on observation of granulomatous inflammation in pleural biopsy tissue. The non-TB pleurisy group included 11 malignant effusions (lung cancer), 14 transudates caused by liver cirrhosis, and 2 empyema. All TB patients were recruited prior to anti-TB chemotherapy treatment and some of them were follow-up after anti-TB chemotherapy treatment for C1q dynamic assays. Heparinized whole blood was collected by venipuncture from the populations mentioned above, broncho-alveolar lavage fluid (BALF) was collected from some active TB patients, and pleural fluid samples were collected from patients with pleural effusion.

**Table 1:** The demographic characteristics of the study populations.

Study cohort	n	Median age in years (range)	Gender (M/F)	AFB-positive	ELISPOT Positive
Microarray set					
TB	9^a^	29.5 (18.0–52.0)	6/3	9	9
LTBI	6	30.3 (25.0–37.0)	3/3	ND	6
HC	6	28.5 (22.0–36.0)	3/3	ND	0
Validation cohort					
TB	164^d^	30.9 (18.0–57.0)	106/58	143	164
LTBI	142	29.4 (18.0–52.0)	117/25	ND^b^	142
HC	45	30.9 (18.0–46.0)	28/17	ND	0
Pleurisy cohort					
TB	36^e^	29.5 (18.0–48.0)	29/7	NA^c^	36
non-TB	27	43.3 (25.0–68.0)	17/10	NA	NA

a These patients were follow-up to 3, 6 months after anti-TB chemotherapy, and gene expression profile were performed by microarrays.

b “ND” indicated not done.

c “NA” indicated not applicable.

d 14 patients were follow-up to 0, 3, 6, 12 months after anti-TB chemotherapy for examine the dynamic change of ClqC level in plasma.

e PBMC and PFMC from the same patient (n = 15) were collected to compared the C1q expression in different tissues.

Specifically, heparinized whole blood was collected to isolate peripheral blood mononuclear cells (PBMC) as described [30]. PFMC and supernatants were separated by centrifuging up to 50 mL pleural fluid at 300×g for 5 min [30]. Broncho-alveolar lavage fluids (BALF) collected from some active TB patients were separated into cell pellet and supernatant following the protocol described elsewhere [31]. Isolated PBMC, PFMC, and broncho-alveolar lavage (BAL) cell pellet were stored at −150°C before RNA extraction. The plasma, supernatant of pleural fluid and BALF were stored at −80°C for C1qC and cytokine measurement. The protocols were approved by the Institutional Review Board of Shenzhen Third People's Hospital. Written informed consent was obtained from all participants.

### Microarray Assays

Total RNA was extracted from the PBMC with Trizol Reagent (Invitrogen, Carlsbad, CA) according to the manufacturer's instructions. Microarray assays were performed at the Shanghaibio Corporation (National Engineering Center for Biochip in Shanghai, China) using the Affymetrix U133Plus 2.0 GeneChip oligonucleotide arrays with 54,675 probes representing 29,255 genes/transcripts (Affymetrix, Santa Clara, CA, USA). Raw data generated from affymetrix Human U133Plus2.0 were processed and normalized by EXPRESSION CONSOLE software (version 1.1.2). All the microarray data had been deposited in GEO database with accession number: GSE54992.

### Validation of C1qA/B/C Expression by qRT-PCR

Total RNA was extracted using QIAamp RNA Mini Kit (Qiagen GmbH, Germany) following the manufacturer's instructions. Purified RNA was reverse transcribed to cDNA using PrimeScript®RT reagent Kit (TaKaRa) according to the manufacturer's protocol. qPCR was performed using SYBR™ Green PCR Master Mix (TaKaRa) in a 20-μl reaction volume. The qPCR was performed under the following conditions using an ABI7500 instrument: 95°C for 10 s, followed by 40 cycles of 95°C for 15 s and 60°C for 35 s. The relative mRNA expression of different genes was calculated by comparison with the housekeeping gene GAPDH using the 2^−ΔΔCt^ method. [32]

### Intracellular Cytokine Staining and Flow Cytometric Analysis

Intracellular cytokine staining was done as previously described [33]. Fresh heparinized whole blood from HC, LTBI or TB was incubated with ionomycin (1 mg/ml; Sigma-Aldrich) and phorbol myristate acetate (50 ng/ml; Sigma-Aldrich) for 2 h, and then incubated with brefeldin A (10 mg/ml; Sigma-Aldrich) for 4 hours at 37°C. Cells were then washed in PBS, and stained with surface mAbs against CD3, CD14 and C1q, (BD Bioscience) fixed with FACS Lysing Solution(BD Bioscience), permeabilized with permeabilizing solution (BD Bioscience), and intracellular stained with mAbs against CD68 (BD Bioscience). Cells were then washed and fixed in 1% paraformaldehyde. Data were acquired on FACS CantoII (BD Biosciences).

### Immunohistochemistry and Tissue Laser Confocal Microscopy

For immunohistochemistry and tissue laser confocal microscopy, lung tissue from patient with tuberculosis by surgery were collected and frozen in liquid nitrogen. Serial 5 μm sections of paraffin-embedded tissue were used for immunohistochemical staining. Briefly, tissues were fixed with acetone/chloroform for 3 minutes, then incubated for 2 hours with monoclonal antibody to mouse CD68 and polyconal antibody to rabbit C1q (ZsBio). Primary antibodies were detected using bioinylated secondary antibodies system (PolinkDS-MR kit, Golden Bridge International Co.) following the manufacturer's instruction. Images were photographed using NanoZoomer Digital Pathology System (Hamamatsu Photonics). For laser confocal analysis, the specific secondary antibodies were used: Alexa Fluor 488 goat anti-mouse, and Alexa 647 anti-rabbit antibody (Invirtrigen). Negative controls were obtained by using isotype-matched primary antibodies. Tissue were stained with Hoechst 33342 (Sigma) before observation. The tissues were observed and imaged using a Zeiss LSM700 laser scanning confocal microscope.

### Detection of C1qC in Plasma and Pleural Fluid Samples

The concentration of C1qC in plasma and pleural fluid was determined by enzyme-linked immunosorbent assay (ELISA) following the manufacturer's instruction (USCN Life Science Inc., Huston, USA).

### Determination of Cytokines Level and Adenosine Deaminase Activity (ADA) in Pleural Fluid

The concentration of proinflammatory cytokines IL-1α, IL-1β, IL-6, and TNF-α in pleural fluid were determined by Luminex liquid array-based multiplexed immunoassays (Luminex Corporation, USA) according to the manufacturer's instruction (Millipore, USA) as described previously [34]. ADA was measured by routine biochemical analysis in the clinical laboratory of Shenzhen Third People's Hospital.

### Statistical Analysis

All statistical tests were performed with Prism 5.0 (GraphPad). The one-way ANOVA Newman–Keuls Multiple Comparison Test was used for statistical analyses to compare the differences among multiple groups. Unpaired *t*-test was used to analyze the difference between two groups. Paired *t*-test was used to analyze the difference between paired samples. Differences were considered significant for *P*<0.05. Receiver operating characteristic (ROC) analysis was performed to determine the power of each candidate biomarker to distinguish TB from HC and TB from LTBI.

## Results

### Active TB is Associated with Increased C1q Levels in Peripheral Blood

**Figure 1:**
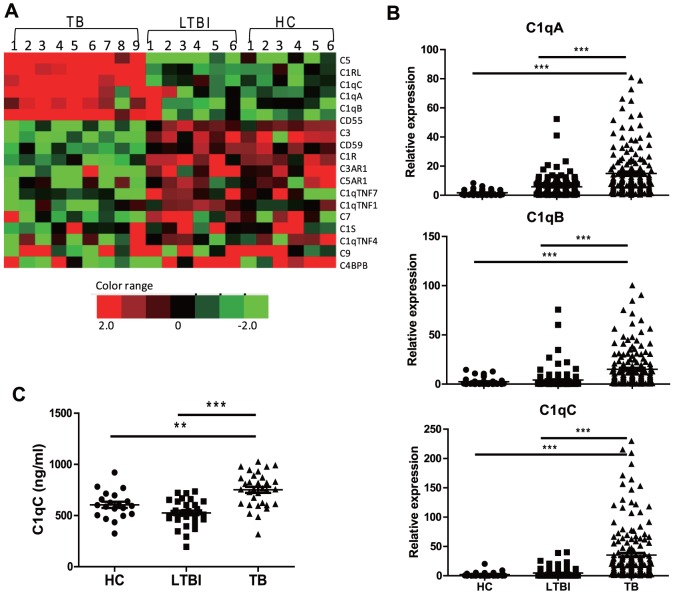
Increased C1qA/B/C expressions in active TB patients. A) Comparison of the transcriptional signature of the complement genes as determined by microarray assays between HC (n = 6), LBTI (n = 6), and TB (n = 9) PBMC samples. Pseudocolors indicate differential expression (red, up-regulation; green, down-regulation; black, no change in expression). B) Expression of C1qA/B/C genes in PBMC from validation cohort 1 consisting of HC (n = 45), LTBI (n = 142), and TB (n = 164) was determined by qRT-PCR. Relative gene expression was normalized to GADPH. C) ELISA of C1qC concentration in the plasma from HC (n = 20), LTBI (n = 32), and TB (n = 32). The means of C1qC concentration in HC LTBI and TB were 583.8±134.3, 525.8±124.0 and 751.4±166.1 ng/ml, respectively. Differences between groups were compared with the one-way analysis of variance followed by Newman-Keuls multiple comparison test; *P* values are indicated. ** *P*<0.01, *** *P*<0.001.

As an initial step for identifying potential biomarkers for diagnosis of active TB, we performed genome-wide transcriptional analysis in PBMC isolated from 9 PTB patients, 6 LTBI and 6 HC. We observed that increased expression of complement genes was associated with active TB (Fig. 1A). In particular, C1qA, C1qB, and C1qC gene expression were significantly increased in TB patients compared to LTBI and HC. This difference was validated by a conventional qRT-PCR assay using the same samples (data not shown) as well as an additional cohort (Fig. 1B). The increase in C1q was also confirmed at protein level (Fig. 1C), with TB patients displaying significantly elevated circulating C1qC protein relative to LTBI and HC.

### Active TB Patients Exhibited Increase in Percentage of C1q-expressing CD14 Positive Cells

**Figure 2:**
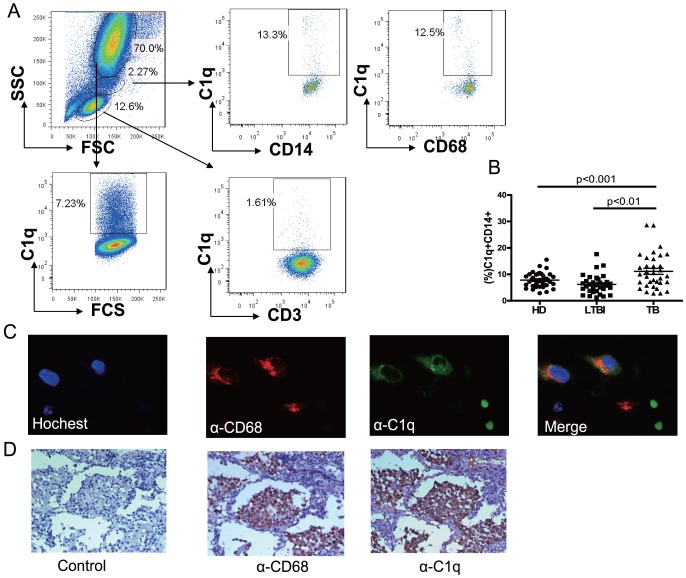
Active TB patients exhibited increase in percentage of C1q-expressing CD14 positive cells. A) Expression of C1q in different cell types. Peripheral blood was stained with anti- CD3, CD14, C1q and CD68 antibody and C1q expression is determined by flow cytometry. B) The percentages of C1q expressing on CD14+ cells from active TB (n = 30), LTBI (n = 30) and HC (n = 30). Differences between groups were compared with the one-way analysis of variance followed by Newman-Keuls multiple comparison test; *P* values are indicated. ** *P*<0.01, *** *P*<0.001. C) Immunofluorescence and D) Immunohistochemistry analysis of C1q expressing in TB patient's lung tissue.

In order to identify which cell types expressed C1q, flow cytometry was performed on peripheral blood of TB patients. Results showed that C1q was mainly expressed in CD14+ and CD68+ monocytes and granulocytes (Fig. 2A). We further explored whether C1q expression on the cell surface was also increased in active TB patients. Peripheral bloods from validation cohort were used to flow cytometric analysis. In agreement with the mRNA and ELISA results, we found that the percentage of C1q-expressing CD14+ cells was significantly increased in active TB patients when compared with HCs and LTBI (Fig. 2B). However, there is no significant difference in granulocyte among three groups (data not shown).

Next, the cellular source of C1q was investigated in TB patient's lung tissue by Immunohistochemistry and immunofluorescence. Results showed that C1q was distributed in cytoplasm and cell surface (Fig. 2C). In addition, C1q is expressed in CD68 positive cells (Fig. 2C&D). These results suggested that macrophages may be the cellular sources of C1q in tuberculosis.

### C1q Expression in Peripheral Blood Correlates with Sputum Smear Positivity in PTB Patients and is reduced after anti-TB chemotherapy

**Figure 3:**
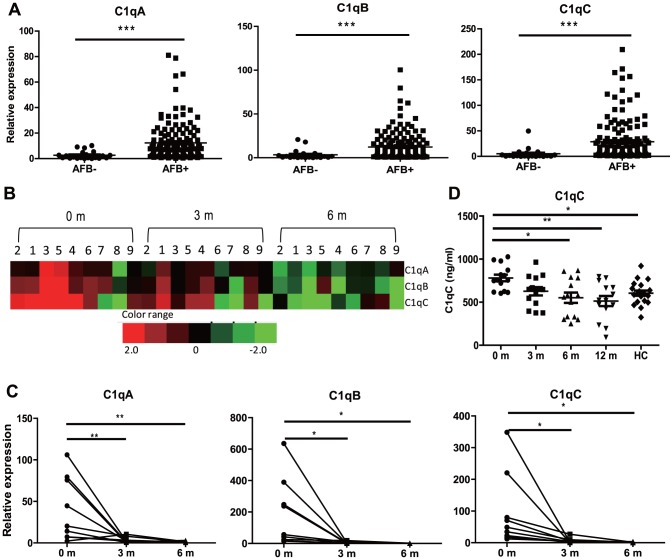
C1q Expression in PBMC is associated with sputum smear AFB positivity and reduced after anti-TB treatment in PTB patients. A) qRT-PCR assay of C1qA/B/C gene expression in PBMCs from sputum smear AFB negative (AFB-, n = 21) and positive PTB patients (AFB+, n = 143). B) Microarray and C) qRT-PCR assay of C1qA/B/C gene expression in PBMC from active PTB patients (n = 9) at 0, 3 and 6 months after initiation of anti-TB treatment. D) ELISA of C1qC concentration in the plasma of PTB patients (n = 14) at 0, 3, 6, and 12 months after initiation of anti-TB treatment. The means of C1qC concentration at 0, 3, 6, and 12 months after anti-TB treatment were 781.2±144.5, 626.3±177.8, 522.1±223.1 and 513.3±220.2 ng/ml, respectively. Differences between AFB- and AFB+ patients were compared with Unpaired t-test; C1q expression at different time points were compared with the one-way analysis of variance followed by Newman-Keuls multiple comparison test; *P* values are indicated. * *P*<0.05, ** *P*<0.01, *** *P*<0.001.

The observation that C1q is increased in active TB and is associated with pulmonary inflammatory conditions suggested that C1q may be involved in the immunopathology of TB and prompted us to investigate the association between C1q expression and severity of PTB. Previous studies have indicated that sputum smear positive PTB patients (AFB+) have more severe lung pathology than those are sputum smear negative (AFB-) as determined by high-resolution computer tomography [35], [36]. We therefore compared mRNA expression in smear AFB+ and AFB− active TB patients (Figure 3A). Importantly, we found that C1qA/B/C mRNA levels were significantly higher in smear AFB+ PTB patients than those AFB− PTB patients.

The results demonstrate that C1q expression is associated with active TB disease and suggest that resolution of infection/inflammation would lead to a reduction in C1q. To test this hypothesis, we monitored C1q expression in the peripheral blood of PTB by microarray analysis before and after anti-TB chemotherapy. As shown in Figure 3B, the levels of C1qA/B/C transcriptional signature determined by microarray declined 3 months after initiation of treatment. This finding was further confirmed by qRT-PCR (Fig. 3C), with C1qA/B/C mRNA expression being reduced to a background level by 3 and 6 months after initiation of treatment. A similar trend was also observed for plasma C1qC protein levels as determined by ELISA (Fig. 3D). This drop in C1q expression in peripheral blood supports the hypothesis that C1q expression is associated with active PTB disease.

### C1q in Peripheral Blood is a Useful Marker for Distinguishing Active TB from LTBI

**Figure 4:**
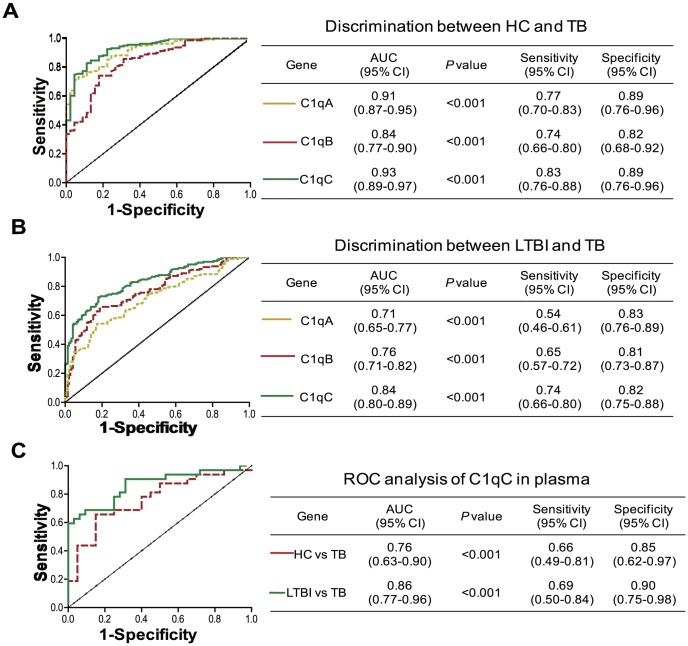
C1q is a useful marker to distinguish active TB patients from HC and LTBI. ROC analysis of the power of C1qA, C1q B, and C1qC mRNA to distinguish A) active TB from HC, and B) active TB from LTBI. C) ROC analysis of the power of C1qC protein levels in plasma from HC, LTBI and TB. AUC, area under curve. 95%CI, 95% confidence interval.

Distinguishing active TB from LTBI remains a challenge in the clinic. The finding that C1q is increased in active TB compared to LTBI samples suggests that C1q could be a useful marker to distinguish active TB from LTBI. To test this hypothesis, ROC analysis was performed to test the power of C1q to distinguish HC/LTBI from active TB. Importantly, we found that C1qA/B/C mRNA expression in PBMC had the power to distinguish TB from HC (Fig. 4A). Among three subcomponents, C1qC exhibited the strongest diagnostic power in discriminating active TB from LTBI (Fig. 4B). In addition, we also found that the C1qC protein levels in plasma also had the power to distinguish TB from HC and TB from LTBI (Fig. 4C)

### Increased C1q Expression at Sites of Active TB Disease Relative to Peripheral Blood

**Figure 5:**
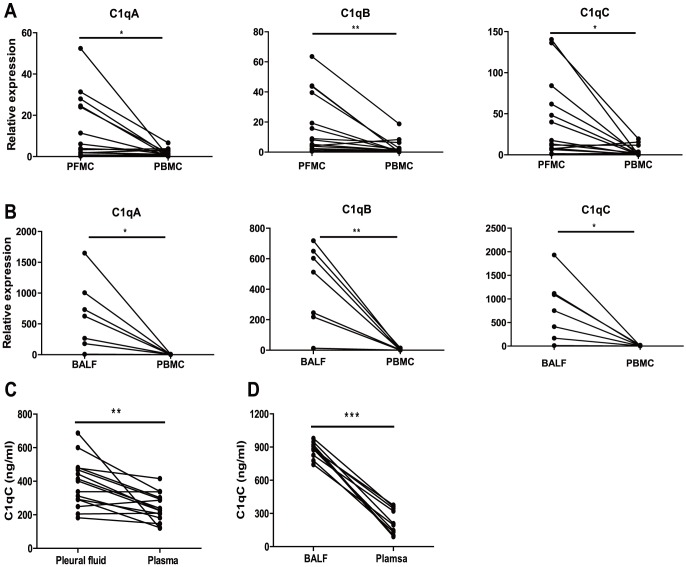
Increased C1q expression in the pleural fluid and BALF compared to matched PBMC from TB patients. A) qRT-PCR assay of C1q A/B/C mRNA levels in PFMC and PBMC from the same TB patient (n = 15). B) qRT-PCR assay of C1q A/B/C mRNA levels in BALF cells and PBMC from the same TB patient (n = 7). C) ELISA of C1qC concentration in pleural fluid and plasma from the same TB patient (n = 15). D) ELISA of C1qC concentrations in BALF and plasma from the same TB patient (n = 7). Differences between groups were compared with paired *t*-test, *P* values are indicated. * *P*<0.05; ** *P*<0.01; *** *P*<0.001.

To compare C1q expression in blood and at the site of infection, we compared C1q expression in pleural fluid with that in parallel peripheral blood of patients with TB pleurisy. As shown in figure 5A, C1q A/B/C mRNA expressions were significantly higher in PFMC than in matched PBMC. To further validate this finding, we compared C1qA/B/C gene expression in BALF with that in paralleled peripheral blood from PTB patients. As seen for the PFMC, C1qA/B/C mRNA expression in cells isolated from BALF was significantly higher than the paralleled PBMC (Fig. 5B). Further, significantly higher C1qC protein level was found in pleural fluid or BALF than in matched plasma (Fig. 5C & 5D). Taken together, these findings indicate that C1q expression is significantly increased at the site of active TB and that this is reflected in peripheral blood.

### Increased C1qC level in Pleural Effusion Distinguishes TB from Non-TB Pleurisy

**Figure 6:**
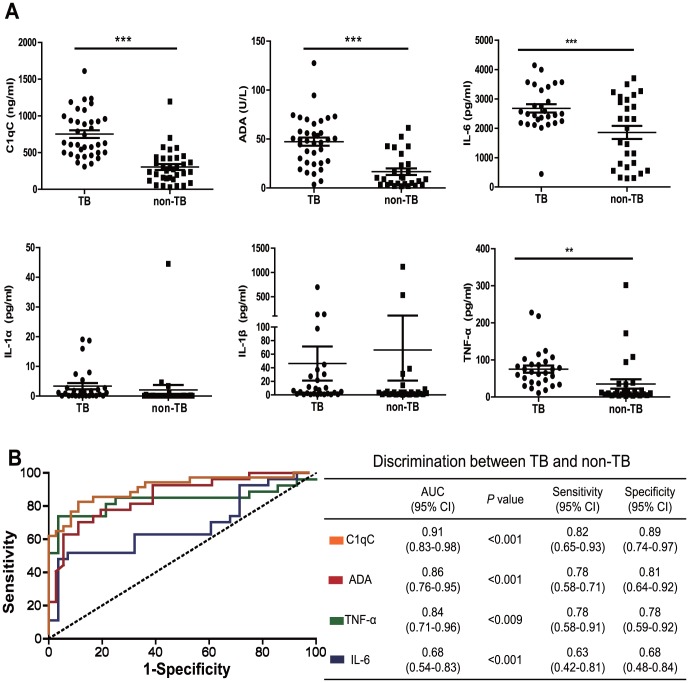
Increased C1qC level in the pleural effusion distinguishes TB from Non-TB pleurisy. A) The levels of C1qC, ADA, IL-6, IL-1α, IL-1β, and TNF-α in pleural fluid from TP patients (TB, n = 36) and non-TB patients (non-TB, n = 34) were determined by Luminex liquid array-based multiplexed immunoassays. Differences between groups were compared with Un-paired t-test, P values are indicated. * P<0.05; ** P<0.01; *** P<0.001. B) ROC analysis of the power of ADA, C1qC, IL-6, and TNF-α in pleural fluid to distinguish TP from non-TB. AUC, area under curve; 95%CI, 95% confidence interval.

To determine whether C1q or local inflammatory cytokines are useful biomarkers for the differential diagnosis of TB pleurisy, we compared the levels of C1qC as well as the levels of proinflammatory cytokines IL-1, IL-6 and TNF-α in pleural fluid of TB and non-TB pleurisy. The protein levels of C1qC, IL-6, and TNF-α were significantly higher in pleural fluid of TB patients than those of non-TB patients (Figure 6A). On the other hand, no significant differences of IL-1α and IL-1β level were found between the two groups of patients (Fig. 6A), suggesting that some but not all proinflammatory cytokines can be used for dissociating TB from non-TB pleurisy.

Next, we compared the power of C1qC, IL-6, and TNF-α to discriminate TB and non-TB pleurisy. As a positive control, we also determined the discriminatory power of ADA, which is elevated in TB patients and is been routinely as a diagnostic marker in the clinics [37]. Consistent with a previous report [28], ADA has the best performance in discriminating TB from non-TB pleurisy (Fig. 6B). Nevertheless, C1qC has the highest discriminatory power relative to IL-6, and TNF-α with a sensitivity of 0.82 (95%CI, 0.65–0.93) and specificity of 0.89 (95%CI, 0.74–0.97) in distinguishing TB from non-TB pleurisy (Fig. 6B).

## Discussion

Two global gene expression microarray analysis studies have previously shown that compared to LTBI and HC subjects, TB patients displayed significantly increased C1q mRNA expression in peripheral blood cells [29], [38]. However, the biological and clinical significance of these findings has up to now not been investigated. In our studies we confirmed using microarray data that active TB was associated with an elevated C1q expression. In addition and expanding upon previous work we validated this difference in expression using qRT-PCR and flow cytometry. We then looked the association further and showed that C1q expression in the blood decreased following anti-TB chemotherapy. We also show here that TB pleurisy was associated with a higher level of C1qC in pleural effusion than was seen in non-TB pleurisy. Together, these findings strongly support the concept that C1q measurement could assist in identifying patients with active TB and also in disease prognosis following treatment.

Recently, several host molecules have been suggested as tools to discriminate active TB from LTBI [39]. A whole-blood 86-transcriptional signature which is dominated by a neutrophil-driven interferon-inducible gene profile has been reported to distinguish active TB from other diseases with a specificity of 83% [40]. In an independent study, Maertzdorf *et al*. identified a unique subset of genes (FCGR1B, CD64, LTF, guanylate binding protein 5 and Granzyme A) that were also capable of distinguishing active TB from LTBI [29]. While our study also found an increase in the expression of some interferon inducible genes as well as FCGR1B and CD64 (data not shown), the ROC analysis revealed that C1qC mRNA expression alone in PBMC can distinguish active TB from HC with a sensitivity of 83% and specificity of 89%, and active TB from LTBI with a sensitivity of 74% and specificity of 82%, respectively. These findings suggest that in addition to interferon-inducible genes, an elevated C1q is strongly associated with active TB disease and could be used to discriminate active TB from LTBI. Novel molecular diagnostic approaches with the combination of these gene signatures may significantly improve our ability to identify active TB cases.

Maertzdorf *et al*. reported an increase in C1qA and C1qB, but not C1qC, in patients with active TB [29]. However, the difference in mRNA expression between active TB and LTBI groups determined by microarray was moderate (2–3 fold) and importantly, its potential in discriminating active TB from LTBI was not evaluated [29]. We found in this current study that C1qC is the most differentially expressed gene among the three C1q components. The reasons underlying the difference are currently unknown. In addition to donor ethnic background, we suspect that the difference in cell populations used (whole blood vs. PBMC) might have contributed to the discrepancy. Compared to whole blood used in the previous study, PBMC preparation used here are known to contain significantly more monocytes and less neutrophil [41]. Experiments are now underway in our laboratory to examine the expression of C1q in various leukocyte populations.

Importantly, our longitudinal study indicated that the level of C1q in PBMC could be used to monitor the efficacy of anti-TB chemotherapy. A previous paper showed that C1q mRNA expression diminished as early as 1 week after treatment [38]. Our kinetic study has extended the finding and demonstrated that C1q mRNA expression progressively declined through the course of treatment and was completely extinguished by 6 months. In contrast, plasma C1q protein took longer and returned to background level by 12 months after treatment. These observations suggested that C1q mRNA analysis may be more sensitive than protein measurement in monitoring the outcome anti-TB treatment.

In a substantial development we found that the C1q level in pleural effusion and BALF of TB patients is significantly higher than that of non-TB patients, suggesting that C1q is highly enriched at sites of active TB disease. It remains unclear however whether C1q is produced locally at the inflammatory sites or systemically in organs like the liver. Nevertheless, the observation raises the possibility that C1q may actively contribute to the regulation of the inflammation in active TB. Indeed, locally synthesized C1q has been reported to act in an autocrine/paracrine manner to control microglial activation and recruitment through regulating the production of the pro-inflammatory cytokines such as IL-1α, IL-1β, IL-6 and TNF-α [42]. Inhibition of inflammation at early stage gives Mtb a breathing space to initiate a productive infection and therefore cause disease [43]. To do this, Mtb has developed strategies to inhibit macrophage activation [43], [44]. For example, it has been recently reported that pathogenic mycobacteria use cell-surface-associated phthiocerol dimycoceroserate lipids to mask underlying pathogen-associated molecular patterns and therefore preventing Toll-like receptor pathway mediated activation of macrophage [44]. In addition, it has been reported that intranasal Poly-IC treatment exacerbates tuberculosis in mice through the pulmonary recruitment of a pathogen-permissive monocyte/macrophage population, which were not fully activated and displayed impaired control of Mtb [45]. On the other hand, macrophage also plays important role in protective immunity against Mtb as the cell is responsible for both killing the bacteria directly and priming immune response by antigen presentation [43]. As C1q also plays a role in facilitating activation of macrophage and maturation of dendritic cells, [24], [26], [27], [28] further investigations are warranted to clarify the exact role of the increased C1q at sites of active TB disease, and if so, how the complement production is regulated.

One limitation of this study is that we did not determine whether C1q expression could be used to differentiate active TB from other non-mycobacterial, acute inflammatory diseases. This is important because similar to IL-6 and CRP, increased C1q level has been reported during other inflammatory conditions [46], [47]. Interestingly, we found that the C1qC level in pleural effusion of TB patients is significantly higher than in non-TB patients (non-inflammatory diseases). It is encouraging that ROC analysis showed that the C1qC level in pleural fluid is better than IL-6 and TNF-α in distinguishing TB from non-TB pleurisy. Nevertheless, to formally establish a role for C1q in differential diagnosis of active TB, we will in future recruit individuals with active TB and non-TB lung disease and compare their C1q levels.

Although in this study we have clearly demonstrated the important value of C1q in diagnosing active human TB, it remains unclear if the molecule plays a role in controlling host resistance and inflammatory response to Mtb. The precise functions of C1q in TB immunopathology and disease progression warrant further investigation.
